# *E. coli* allantoinase is activated by the downstream metabolic enzyme, glycerate kinase, and stabilizes the putative allantoin transporter by direct binding

**DOI:** 10.1038/s41598-023-31812-4

**Published:** 2023-05-05

**Authors:** Irina A. Rodionova, Ali Hosseinnia, Sunyoung Kim, Norman Goodacre, Li Zhang, Zhongge Zhang, Bernhard Palsson, Peter Uetz, Mohan Babu, Milton H. Saier

**Affiliations:** 1 Department of Molecular Biology, Division of Biological Sciences, University of California at San Diego, La Jolla, CA 92093 USA; 2 Department of Bioengineering, Division of Engineering, University of California at San Diego, La Jolla, CA 92093-0116 USA; 3 Department of Biochemistry, University of Regina, Regina, SK S4S 0A2 Canada; 4 Center for the Study of Biological Complexity, Virginia Commonwealth University, Richmond, VA 23284 USA; 5 College of Food Science and Engineering, Ocean University of China, Yushan Road, Shinan District, Qingdao, 266003 China; 6 Department of Pediatrics, University of California San Diego, La Jolla, CA 92093 USA; 7 Novo Nordisk Foundation Center for Biosustainability, Technical University of Denmark, 2800 Lyngby, Denmark

**Keywords:** Biochemistry, Cell biology, Microbiology, Molecular biology, Systems biology

## Abstract

Allantoin is a good source of ammonium for many organisms, and in *Escherichia coli* it is utilized under anaerobic conditions. We provide evidence that allantoinase (AllB) is allosterically activated by direct binding of the allantoin catabolic enzyme, glycerate 2-kinase (GlxK) in the presence of glyoxylate. Glyoxylate is known to be an effector of the AllR repressor which regulates the allantoin utilization operons in *E. coli*. AllB has low affinity for allantoin, but its activation by GlxK leads to increased affinity for its substrate. We also show that the predicted allantoin transporter YbbW (re-named AllW) has allantoin specificity and the protein–protein interaction with AllB. Our results show that the AllB-dependent allantoin degradative pathway is subject to previously unrecognized regulatory mechanisms involving direct protein–protein interactions.

## Introduction

Allantoin is abundant in bacteria, fungi, animals and plants as an intermediate of purine degradation via uric acid^1^. Uric acid accumulation and excretion in humans is related to the pathogenesis of gout. The presence of allantoin metabolic genes in *Klebsiella pneumoniae* is connected with liver abscesses caused by this bacterium^2,3^. Moreover, AllS, the allantoin operon transcriptional activator, was found to be a virulence determinant^2,3^. *Escherichia coli* is well known for uropathogenic conditions and other types of infections in humans, and it is a primary cause of infant mortality world-wide.

**Figure 1:**
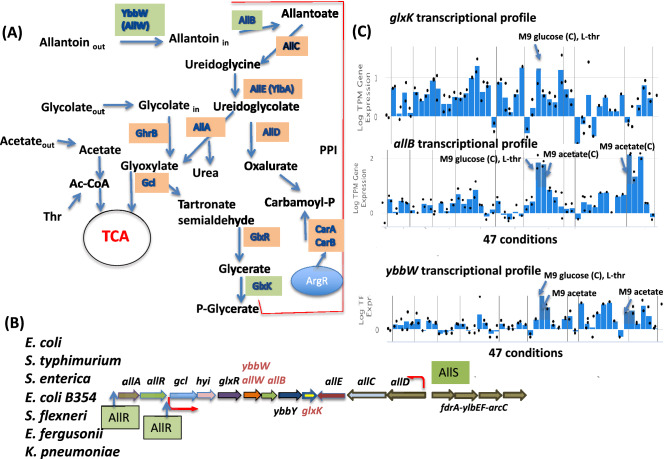
The allantoin degradation pathway in *E. coli.* (**a**) The pathway with metabolites, enzymes (boxed), and reactions (arrows between the substrates and products). Abbreviations: the allantoin permease (AllW), allantoinase (AllB), allantoate amidohydrolase (AllC), ureidoglycine hydrolase (AllE), ureidoglycolate lyase (AllA) and ureidoglycolate dehydrogenase (AllD). Other abbreviations are described in the text. (**b**) The allantoin utilization gene cluster in *E. coli* (genes encoding analyzed proteins are in green boxes). The promoters are marked with red arrows and indicate the direction of transcription. (**c**) The transcriptomic data for *glxK*, *allB* and *ybbW* genes are from iModulonDB. The data are shown for 47 conditions. TPM indicates “transcript per million”. Transcript level for M9 glucose with L-threonine as supplement and M9 with acetate as carbon source are marked by arrow.

Extracellular allantoin is converted to glyoxylate in five steps. The first step involves a putative allantoin transporter YbbW (TC# 2.A.39.3.8), which belongs to the Nucleobase:Cation Symporter Family (TC# 2.A.39), all characterized members of which are transporters. *E. coli* YbbW is 39% identical and 63% similar throughout its entire length to the functionally characterized Gram-positive bacterial (*Bacillus subtilis*) allantoin transporter, PucI^4^. Following entry into the cytoplasm, four enzymes function in allantoin metabolism: (a) metal ion-dependent allantoinase (AllB), producing allantoate^5^, (b) allantoate amidohydrolase (AllC), yielding ureidoglycine^6^, (c) AllE (YlbA), producing ureidoglycolate^7^ and (d) ureidoglycolate lyase (AllA), generating glyoxylate^8^ (Fig. 1a). Ureidoglycolate is metabolized via two pathways. First, it is converted to 2-phosphoglycerate in 4 steps involving ureidoglycolate lyase (AllA), glyoxylate carboligase (Gcl), tartronate semialdehyde reductase (GlxR) and glycerate 2-kinase (GlxK), a pathway that is used for energy metabolism and is regulated by three transcriptional regulators RutR, AllR and AllS^7,9,10^ (Fig. 1b). Second, ureidoglycolate can be metabolized into oxalurate and carbamoyl-phosphate (carbamoyl-P) via ureidoglycolate dehydrogenase (AllD) and oxamate transcarbamoylase (unknown gene), leading to the production of three NH_3_ molecules (Fig. 1a). Carbamoyl-P is a building block for de novo synthesis of pyrimidines and arginine and is optionally synthesised by carbamoyl-phosphate synthase (CarAB) from L-glutamine, ATP and bicarbonate.

The genes encoding the proteins of allantoin utilization are organized in 4 operons within a single gene cluster in *E. coli,* (a) *allAR*, (b) *gcl-hyi-glxR-ybbW-allB*-*ybbY-glxK,* (c) *allDCE,* and (d) *fdrA-ylbEF-arcC* (Fig. 1b). ArcC, carbamate kinase^11^, is involved in the last step of allantoin utilization as a nitrogen source. FdrA and YlbF are proteins of unknown function, although FdrA has been suggested to be an oxamate-CoA ligase^12^. Two regulators, AllR and AllS, influence the transcription of the genes in the allantoin metabolic pathway in response to cytoplasmic allantoin and glyoxylate concentrations, respectively^9,13^. The allantoate utilization genes of the *gcl* operon have been shown to be regulated by the AllR repressor while the *allDCE* operon is regulated by the AllS activator (Fig. 1b)^9,14^. A RutR binding site has been found upstream of the *gcl* operon^13^. RutR is a global regulator of genes concerned with the synthesis of pyrimidines and arginine as well as the degradation of purines^10^, and it plays a role in the activation of the *carAB* P1 promoter.

The glyoxylate branch point in allantoin utilization may provide a connection between purine catabolism and amino acid recycling through transamination between the unstable ureidoglycine and glyoxylate. Such an aminotransferase (PucG) has been biochemically characterized in *B. subtilis*^15^ but not in *E. coli.*

We have collaboratively investigated the existence of a network of protein–protein interactions (the interactome) in *E. coli*^16,17^*.* The work presented in this communication describes allosteric regulation involving direct protein–protein interactions in the allantoin utilization pathway. Proteins found to interact with the glycerate kinase, GlxK, are involved in allantoin degradation: YbbW and AllB are both encoded by genes adjacent to one another in the *gcl* operon, and a hypothetical transporter, YbbY, is also encoded within the same operon^16^ (Fig. 1b). An additional interaction involves AllB and YbbW^16^, and the latter protein was also found to interact with a YdaN Zn^2+^ efflux transporter^18^. Interestingly, AllB requires a relatively high concentration of zinc for activity^5,19^.

When the cytoplasmic concentration of the AllR repressor’s effector, glyoxylate, increases, and the AllR-dependent *allS* gene (activator) is derepressed, the *allDCE* operon becomes activated. This may allow utilization of an alternative route of allantoin degradation through a carbamoyl-P intermediate. Interestingly, interactions of AllC with de novo pyrimidine synthesis enzymes, PyrC and PyrB, have been detected^16^. Two other proteins from the same network that potentially interact with AllC are GlxR (2-hydroxy-3-oxopropionate reductase) and CarA (a carbamoyl-P synthase subunit)^16^. Although the pathway of allantoin degradation and recycling is known, how its activity is controlled is still unclear.

Here we characterize the allantoin transport activity, catalyzed by YbbW, which we have renamed as AllW, in the presence and absence of AllB. Stability and activity of the AllW transporter were both lost in the absence of AllB. An immunoprecipitation assay showed that no intact AllW protein could be detected following its overexpression in the absence of interaction with AllB, but a large peptide (30 KDa), derived from AllW, was present, providing evidence for proteolysis. An immunoprecipitation assay showed that the presence of glyoxylate stabilizes the interaction between AllW and AllB. AllW-His could be recovered in an intact form when binding AllB, but the same sample in the absence of AllB showed only the 30 kDa sized fragment of AllW.

AllB has an extremely high *K*_*m*_ (more then 15 mM)^5,19^ for its substrate, allantoin, and the enzyme depends on Zn^2+^ or Mn^2+^ for activity, even during overproduction in the cell, both in *E. coli*^5^ and in *Salmonella enterica*^20^. We also found that GlxK, glycerate 2-kinase, allosterically activates AllB by reducing its K_m_ in the presence of glyoxylate. An immunoprecipitation assay showed that the presence of glyoxylate stabilizes the interaction between GlxK and AllB. The concentration of glyoxylate in the cytoplasm reflects 2-carbon utilization as an intermediate in the Krebs cycle/glyoxylate shunt.

## Results

### AllB is essential for the transport of allantoin

To measure allantoin uptake in *E. coli*, we utilized gas chromatography coupled with mass spectrometry (GC–MS), to measure cellular allantoin. The samples were derivatized with trimethylsilyl (TMS), producing allantoin TMS derivatives. The m/z spectrum for standard allantoin has the highest peak for the fragment with 331 m/z at the retention time 8.05 min corresponding to the published peak for allantoin detection (Fig. S1A)^21^. The potential allantoate standard was derived with the incubation of allantoin in the assay mixture with AllB and was very small as detected by GC–MS. We decided to measure the chromatogram for the most abundant peak for allantoin 331 m/z for the cell extracts derived from the cells used for the allantoin uptake assay without metal ions added to prevent AllB conversion of allantoin, and as expected, the same retention time peak at 8.05 min and mass spectrum was observed for the extracts (Fig. S1B). The m/z spectrum at retention time 8.05 min for the allantoin detected in the cell extract is shown in Supplementary Fig. S1C, and the chromatogram for m/z is shown in Fig. S1A. The allantoate peak increase during uptake was not detected in the cell extracts.

To prevent (1) catabolic degradation of allantoate, and (2) any additional protein–protein interaction effects the Δ*allC* gene was constructed in three backgrounds: wild type (WT) cells a Δ*allB* mutant (AW12) and a Δ*allW* mutant, yielding strains: AW11, AW12, AW13, respectively (Table 1) as described in Materials and Methods. AllC is the enzyme that acts on allantoate and produce ureidoglycine (Fig. 1).

**Table 1:** Strains and primers used in this study.

Strains	Genotype or description	Reference or source
BW25113	Wild type, *lacI*^q^ *rrnB*_T14_ Δ*lacZ*_WJ_ Δ*hsdR*514 Δ*araBAD*_AH33_ Δ*rhaBAD*_LD78_	Wanner^22^
BW-RI	BW25113 constitutively expressing *lacI* and *tetR*	Levine^23^
Δ*allW*	Δ*allW* in BW25113	This study
Δ*allB*	Δ*allB* in BW25113	This study
AW11	Δ*allC* in BW25113	This study
AW12	Δ*allW* Δ*allC* in BW25113	This study
AW13	Δ*allB* Δ*allC* in BW25113	This study

Name	Sequence	Use
allW1-P1	agaaaactattccagcaacgcggctatagcgaagatctattgccgaaaactgtgtaggctggagctgcttcg	Chromosomal *allW* deletion
allW2-P2	ttgctctcctgttttttctgctgttgtacgtttctttaataaggcgtaggcatatgaatatcctccttagttc	Chromosomal *allW* deletion
allB1-P1	gaaaacgaagctcgcgttgtagatatcgccgttaaaggcggaaaaattgcgtgtaggctggagctgcttcg	Chromosomal *allB* deletion
allB2-P2	caatgtcgtaaatcacatcaccacgtaagatggttttcgtgatacgcgcgcatatgaatatcctccttagttc	Chromosomal *allB* deletion
allC1-P1	ccgtcaagctatagaagaaacgctgccctggctttcctcttttggcgctgtgtaggctggagctgcttc	Chromosomal *allC* deletion
allC2-P2	cttcggcaaggtcggtaatattggtgcgttccgccgggttatggctgatcattccggggatccgtcgacctg	Chromosomal *allC* deletion

**Figure 2:**
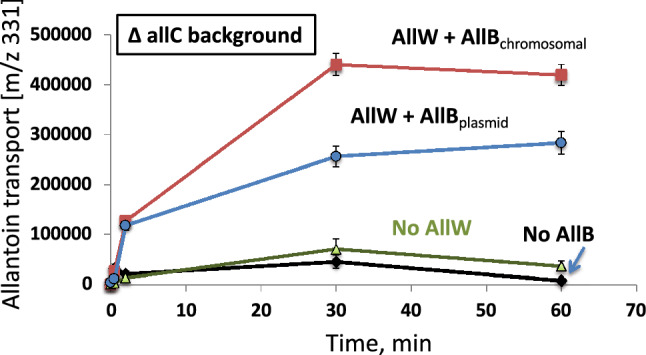
AllB activates AllW-mediated allantoin transport. All strains used in this experiment were deleted for the *allC* gene (*ΔallC*). Squares and circles show the transport kinetics obtained for AllW in the presence of AllB in a *ΔallB* genetic background, expressed from the chromosome and from a plasmid, respectively. This shows complementation of the AllB stabilizing function. Higher transport activity is observed when the adjacent *allW* and *allB* genes are expressed together within the same operon and may result if complex formation occurs during or soon after synthesis, or if a stoichiometric relationship is required for maximal AllW activity.

As shown in Fig. 2, no allantoin uptake was detected in either the AW12 (Δ*allC*–Δ*allB)* or the AW13 (Δ*allC*–Δ*allW*) mutant. However, the allantoin transport activity was restored in the AW12 strain by complementation of the cells with an *allB* overexpressing plasmid, allowing restoration of allantoin uptake activity (Fig. 2).

### Direct binding of AllB to AllW is stabilized by glyoxylate

**Figure 3:**
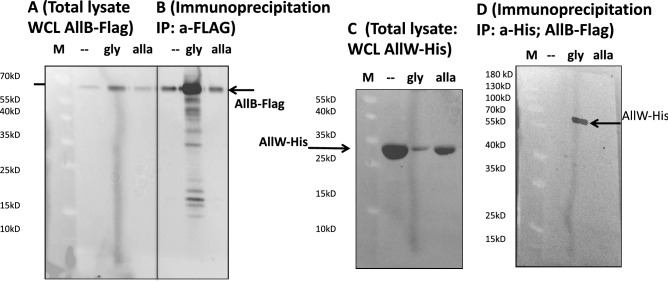
AllB binds to and stabilizes AllW. FLAG-tagged AllB (AllB-Flag) was detected in total lysates (**a**) and immunoprecipitates (**b**), in the presence of glyoxylate (gly) or allantoin (alla), respectively. AllB-Flag was precipitated using anti-Flag-tag coated magnetic beads and then detected with anti-Flag-tag antibodies and ECL (see Methods). (**c**) In the absence of AllB, only the 30 kDa fragment of AllW-His_6_ was detected in the total lysate (using anti-His-tag antibody). (**d**) Intact AllW-His_6_ (54 kDa) was recovered by co-immunoprecipitation (Co-IP) with anti-Flag-tag (AllB) antibodies, only in the presence of glyoxylate. Molecular mass markers (kDa) are indicated on the left side of all figure panels. (**a–c)** full blots are present in the Supplemental Mat. Figs. S3, S4.

To examine the AllB-AllW interaction in vivo, we expressed AllW-His from a plasmid, and AllB-Flag using its native chromosomal promoter (Fig. 3). The intact AllW-His transporter (54 kDa) was detected in complex with AllB-Flag by immunoprecipitation. By contrast, a large fragment (30 kDa) of AllW was identified in a cell lysate when AllB was absent (Fig. 3C).

Flag-tagged AllB was detected using anti-Flag-tag antibody in a total lysate (Fig. 3a) and after immunoprecipitation (Fig. 3b) in the presence or absence of glyoxylate and allantoin. The detection of His-tagged AllW without and with Flag-tagged AllB is shown in Fig. 3C and D, respectively. Here, the Flag-tag was introduced into the chromosomal *allB* gene encoding AllB. In this experiment the AllW-His fragment of 30 kDa was detected when AllB was not present in the insoluble fraction after cell lysis (Fig. 3C). The intact AllW-His (54 kDa) was observed when AllB-Flag was immunoprecipitated from glyoxylate treated cells with anti-Flag-tag antibody but not in glyoxylate untreated or allantoin-treated cells (Fig. 3D), implying that the physical association is enhanced by the presence of glyoxylate.

### Allosteric activation of AllB by GlxK, and AllB-GlxK direct binding

The AllB protein was overproduced in *E. coli* in the presence of ZnSO_4_ (AllB_Zn). Also, His-AllB was produced in the presence of MnSO_4_ (AllB_Mn) and purified to near homogeneity (Fig. S2)^24^. AllB_Mn was able to form a complex with GlxK in the presence of glyoxylate as shown by native gel electrophoresis (Fig. S2, lines 1, 2, respectively).

**Figure 4:**
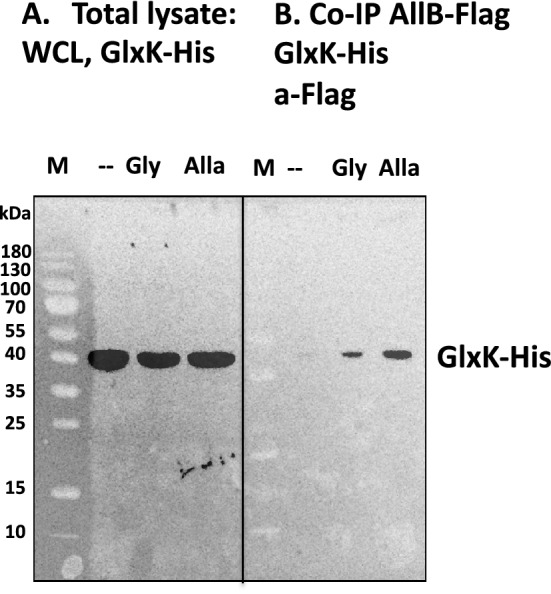
AllB and GlxK interact in the presence of glyoxylate and allantoin. The interaction of His-tagged GlxK and FLAG-tagged AllB in the presence of glyoxylate or allantoin is shown by immunoblotting. (**a**) Detection of GlxK-His (41 kDa) using anti-His-tag antibody following growth in the absence or presence of allantoin (alla, 2 mM) or glyoxylate (gly, 0.7 mM) in *E. coli* DY330 cell extracts. (**b**) Immunoprecipitates of AllB-FLAG in the absence or presence of glyoxylate or allantoin were probed with GlxK-His anti-His-tag antibody. Molecular mass markers (kDa) are indicated on the left side.

For the immunoprecipitation assay, showing the AllB-GlxK interaction, AllB was labelled with a Flag-tag, and the His-tagged GlxK was overproduced from the ASKA collection (Fig. 4a,b). Consistent with this observation, the immunoprecipitants of chromosomally integrated AllB-FLAG showed binding with His-tagged GlxK (overproduced from the ASKA collection) in the presence of glyoxylate and allantoin, but not in untreated cells (Fig. 4).

**Figure 5:**
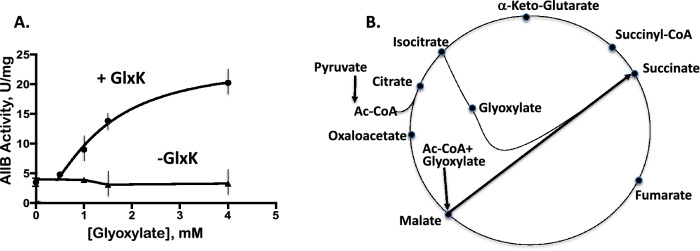
GlxK and glyoxylate synergistically activate AllB. (**a**) the activity of AllB_Zn was measured as a function of the glyoxylate concentration (with 2 mM allantoin) in the presence or the absence of 0.05 μM GlxK, pre-incubated with AllB for 1 h, units are presented in μmoles/min/mg, protein. (**b**) The glyoxylate shunt for 2-carbon source utilization is shown.

Next, we examined the effect of glyoxylate on AllB_Zn activation in the presence of 0.3 μM GlxK (Fig. 5a). Maximal AllB activity was approached at 2–3 mM glyoxylate. The K_m_ for allantoin in the absence of GlxK and glyoxylate was more than 15 mM, corresponding to the published data^5^. We detected that in the presence of GlxK and 1 mM glyoxylate, it decreased to 2.8 ± 0.4 mM. While glyoxylate was required for AllB activation by GlxK, this concentration of glyoxylate (< 2 mM) is probably physiologically relevant when allantoin is rapidly metabolized and/or when the glyoxylate shunt is operative^25^.

The AllB kinetics were also measured for the His-tagged purified protein, overproduced in the presence of 2 mM MnSO_4_ (AllB_Mn)_._ AllB_Mn (15 nM) and the GlxK protein were pre-incubated for 30 min in the assay mixture. The activating effect of GlxK on AllB_Mn in the presence and absence of 0.9 mM glyoxylate is shown in Table 2. Allosteric activation of AllB_Mn by GlxK in the presence of glyoxylate reduced the *K*_*m*_ over threefold. However, activation of AllB_Mn by GlxK and glycolate was much weaker (Table 2, Fig. S5). The presence of glyoxylate as intermediate of glyoxylate shunt suggests that specific growth conditions can activate AllB (Figs. 1, 5b).

**Table 2:** Kinetic parameters of AllB_Mn with respect to allantoin in the presence and absence of GlxK and glyoxylate or glycolate.

	AllB_Mn GlxK, Glycolate, 1 mM	AllB_Mn GlxK, Glyoxylate, 0.9 mM	AllB_Mn No GlxK Glyoxylate, 0.9 mM
*V*_*max*_	378 ± 140	466 ± 99	246 ± 32
*K*_*m,mM*_	7.3 ± 4.0	3.8 ± 0.9	5.8 ± 1.2
*V*_*max*_*/K*_*m*_	51	122	42

The V_max_ is given as U/mg protein at 37 °C, where units are defined as μmol/min.

### Metal ion dependency of GlxK

The effects of a metal ion present in the assay mixture (Mn^2+^ or Mg^2+^) on GlxK activity was measured. GlxK kinase kinetics revealed affinity for glycerate with a *K*_*m*_ of 85 μM in the presence of 10 mM MgSO_4_ is shown in Table 3 and Fig. S6, and kinetic parameters, calculated using Prizm 7, are presented in Table 3. Addition of 0.2 mM MnSO_4_ to the glycerate phosphorylation assay mixture decreased the *K*_*m*_ for glycerate. The GlxK kinetics revealed higher affinity for glycerate in the presence of MnSO_4_ than of MgSO_4_, and an effect of the former ion on the regulation of AllB activity was shown. Then, by measuring the GlxK kinetics in the presence and absence of 0.2 mM MnSO_4_, we suggest that the metal ions are important for the enzymatic activity.

**Table 3:** Kinetic parameters of GlxK with respect to glycerate in the presence and absence of 0.2 mM MnSO_4_.

	MgSO_4_ 10 mM	MgSO_4_ 10 mM MnSO_4_ 0.2 mM
*V*_*max*_	4.6 ± 0.3	3.7 ± 0.3
*K*_*m*_,* μM*	85 ± 9	44 ± 2

The V_max_ is given as U/mg protein at 37 °C, where units are defined as μmol/min.

### Model for docking the AllB-GlxK interaction

**Figure 6:**
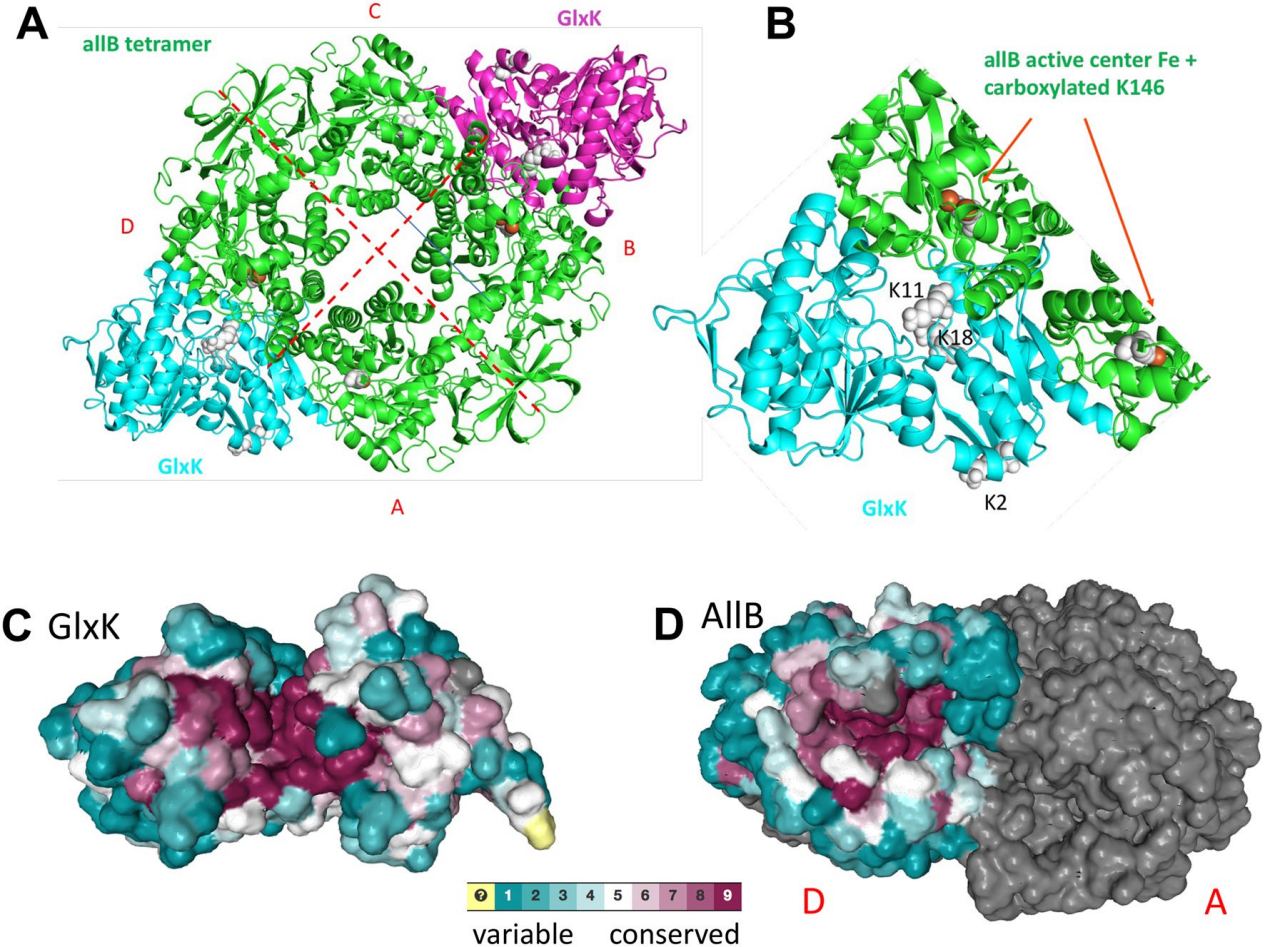
Docking model showing possible binding site of GlxK in the inter-subunit clefts of the tetrameric AllB. (**a**) Model using the tetrameric structure of AllB (PDB: 3E74:A) and GlxK (PDB: 3CWC:A). (**b**) Details from A, showing the iron ion in the active site of AllB and the carboxylated K146. (**c**), (**d**) Consurf models of GlxK and AllB, respectively, with variable (blue) and conserved (red) regions indicated. The gray half in (**d**) represents one of two subunits in a dimer, corresponding to subunit D in panel (**a**).

We used the available structures of the *Salmonella* GlxK (PDB: 3CWC) and the tetrameric *E. coli* allantoinase (AllB) (PDB: 3E74) to generate a model of the most likely configuration of these interacting proteins (Fig. 6). Interfacial residues were calculated using HADDOCK and the structure of the predicted complex is shown in Fig. 6a and summarized in Table 4. Interestingly, the best HADDOCK model predicts that GlxK interacts with AllB near the interface of two homodimeric subunits (A/D and B/C, Fig. 6a). Five GlxK residues (D328, E331, V332, H334, and Y336) are predicted to form interactions with both subunits of AllB.

**Table 4:** Interacting residues for the proposed GlxK–AllB interaction.

Interface	Interacting residues
GlxK–AllB:A/C	GlxK (cyan) 328, 331, 332, 334, 336, 337, 338, 339, 340, 341, 342, 343, 364, 367, 368, 375, 377, 378, 379, 380 AllB:A/C (light green) 128, 129, 165, 166, 167, 168, 191, 194, 195, 198, 201, 202, 226, 230, 318, 319, 320, 323, 330
GlxK–AllB:D/B	GlxK (cyan) 296, 299, 300, 326, 328, 329, 330, 331, 332, 333, 334, 336, 345, 350, 351, 352, 353 AllB:D/B (dark green) 128, 129, 131, 148, 149, 163, 165, 166, 167, 168, 170, 189, 191, 222, 226, 230, 320

N-terminal lysine residues 11 and 18 in GlxK are proximal to the active site in AllB, shown as white spheres (Fig. 6b). Each active site contains a pair of iron atoms and a carboxylated lysine (AllB K146; red and white spheres, respectively, in Fig. 6b). The area surrounding the ligand-binding cleft forms the bulk of the interface of GlxK. The AllB interface involves both the primary subunit A and the neighbouring AllB subunit D, although an active-site proximal binding site is also present (bottom left), as well as an electrostatic patch, which only involves the primary subunit A.

While the active centre of GlxK is highly conserved (Fig. 6c), its surface shows only a few moderately conserved residues (e.g. S343, N361, N368 in PDB: 3CWC). Similarly, the interacting surface of AllB is relatively poorly conserved (Fig. 6d), suggesting that the interaction with AllB is not generally conserved in bacteria, and thus may have evolved recently and is less specific. However, the most striking aspect of the model is that both GlxK and AllB appear to interact with those surfaces that also include the entrances to their active sites (Fig. 6c,d). This suggests that the interaction may increase access to the active sites through a conformational change.

## Discussion

In this study we demonstrated protein–protein interaction-dependent regulation of allantoin transport in *E. coli* involving AllB. In the *E. coli* genome, *allB* is encoded next to *ybbW* (the gene we renamed as *allW*, (Fig. 1b), shown to be essential for allantoin transport in this paper. The intact AllW transport protein was shown to rely on the presence of AllB for activity, suggesting that AllB could prevent proteolysis of AllW. We expressed the *allW* gene with and without the *allB* gene and found full length AllW only when AllB was present with glyoxylate. A large AllW-derived peptide (30 KDa) was identified in precipitates of crude extracts without AllB, which is consistent with our physical and genetic data (Fig. 3). These observations lead to the conclusion that the maintenance of a functional AllW depends on its binding to AllB in the presence of glyoxylate, suggesting that glyoxylate stabilizes the physical association between AllW and AllB.

Allantoin can be used as a nitrogen source by *E. coli* under anaerobic conditions^9^, and glyoxylate is an inducer of the AllS-activated *allDCE* operon. We found that GlxK allosterically activates AllB in the presence of glyoxylate, decreasing the K_m_ for allantoin. The high K_m_ in the absence of GlxK possibly renders GlxK essential for efficient allantoin utilization^26^.

Glyoxylate is utilized through a glycolate/glyoxylate interconversion pathway and is an intermediate in the carbon-conserving glyoxylate shunt pathway (Fig. 5). The glyoxylate shunt is utilized whenever 2-carbon compounds are carbon sources, preventing loss of CO_2_ in the full tricarboxylic acid cycle. We found an increase of *gcl* operon transcription under carbon starvation conditions with glycolate as a carbon source in M9 medium (data not shown). The mRNA for the genes *hyi, glxR, gcl, allB, allA,* and *allW* increased in the adapted strain for the utilization of D-arabinose as carbon source, compared to M9 glucose growth for strain MG1655, suggesting an increase of glyoxylate concentration when D-arabinose is the carbon source^27^.

Poor growth of a *glxK*^*−*^ mutant on allantoin as the nitrogen source has been reported, but an explanation was not available^28^. The allantoin utilization pathway provides an additional molecule of glyoxylate that can feed into the shunt pathway, and this explains the AllB activation in response to the intracellular glyoxylate concentration. Bacterial gene cluster analysis revealed that glyoxylate utilization genes are often present in the context of allantoin utilization (data not shown). Possibly, utilization and functionality of the pathway converting allantoin to glyoxylate are important when 2-carbon sources such as glycolate and acetate are being utilized.

The work described here expands our understanding of how the purine degradative pathway in *E. coli,* and presumably many other bacteria, is regulated. Previous studies revealed the occurrence of complex transcriptional regulation as well as the allosteric regulation of certain enzymes in this pathway by specific metabolites (see Introduction). However, our studies reveal for the first time that direct protein–protein interactions are important for the regulation of allantoin metabolism. Two such interactions were of specific note. First, the AllB binds to the allantoin uptake porter, AllW, apparently with high affinity. Moreover, without this interaction, AllW is rapidly degraded in vivo. By contrast, allantoinase exhibits low affinity for its substrate, allantoin, but the binding of GlxK activates AllB by lowering its substrate K_M_ to a physiologically relevant value. Since GlxK is an essential enzyme for allantoin utilization in *E. coli*, this activity enhancement may contribute to its essentiality.

Our data suggest that protein–protein interactions provide an important mechanism of metabolic control. In this case, we see how one protein can protect another when bound, and that a downstream enzyme in a pathway can activate a key upstream enzyme by increasing its substrate affinity, accelerating the rate of substrate degradation. In this case, GlxK may be a sensor for the level of the product of metabolism, glyoxylate. If so, it not only functions as a regulatory protein, it also serves as a sensor of the cellular metabolic state. Further studies are likely to reveal many more protein–protein interactions in the regulation of metabolism in all living cells. Elucidation of these interactions is expected to reveal complex networks interrelating many aspects of cellular physiology^16^.

## Methods

### AllB and AllW protein overproduction

Recombinant genes were overexpressed in *E. coli*. Cells with *allW* or *allB* alone, or with *allB* plus *allW,* were grown in LB medium (1 L) at 37 °C with 0.2 mM ZnSO_4_ and harvested after 4 h of rotation at 250 rpm. The AllB-His (ASKA overexpression library) protein was produced in the presence of 0.1 mM CoCl_2_ or 2 mM MnSO_4_ at 30 °C and induced by addition of 0.2 mM IPTG when the culture reached OD_600_ = 0.2. Cells were harvested after 18 h and resuspended in 20 mM HEPES buffer, pH 7, containing 100 mM NaCl, 2 mM β-mercaptoethanol and 0.03% Brij 30, with or without 2 mM phenylmethylsulfonyl fluoride. Cells were lysed by incubation with lysozyme (1 mg/ml) for 30 min at 4 °C, followed by a freeze–thaw cycle and sonication. The pellets were collected after lysis, sonication, and centrifugation at 14,000 rpm for 25 min, and the insoluble fraction was resuspended in At-buffer 0.1 M Tris–HCl, pH 8.0, 0.5 M NaCl, 5 mM Imidazole, 0.3% Brij, β-mercaptoethanol, described in^24,29^ and containing 7 M urea and 0.03% Brij 30. Inclusion bodies were dissolved, and after sonication and centrifugation, they were analyzed. Protein size, expression level, distribution between soluble and insoluble forms, and extent of purification were monitored by SDS-PAGE. Protein concentrations were measured using the Bradford assay kit (Bio-Rad). Recombinant glycerate kinase (GlxK), containing an N-terminal His_6_ tag, was overexpressed in *E. coli* and purified as described^16^. The buffer used for GlxK purification was changed to At-buffer, pH 8, by dialysis.

### GC–MS allantoin determination

For the determination of allantoin uptake, AW11, AW12, and AW13 deletion mutants were constructed (see below and Table 1). These mutant strains were grown at 37 °C in LB medium with allantoin as supplement and were then washed twice with M9 medium by centrifugation at room temperature. The uptake of allantoin was measured using freshly grown washed cells by incubating with 1 mM allantoin for different time periods in PBS buffer, supplemented with glucose. At various times, 1.5 ml of the resulting cells (density, OD_600nm_ = 1) was collected and washed by centrifugation, and the pellets were collected and frozen. The cells were resuspended in 0.2 ml acetonitrile/methanol/water, 40/40/20, and the internal standard, norvaline, was added. After freezing and thawing, the cell debris was removed by centrifugation, and the supernatant was transferred to a new tube. The vacuum dried supernatant was dissolved in pyridine and was derivatized using the MS-TFA reagent as previously described^30^, creating trimethylsilyl (TMS) derivatives of allantoin before analyzing the sample by GC–MS. The identification and quantification of allantoin were conducted at m/z 331 for cells collected after 1/2, 2, 5 and 30 min incubations and normalized using norvaline as an internal standard for efficiency of derivatization. The allantoin standard was measured by the same method using GC–MS.

### Isolation of *allW, allB* and *allB–allW* mutants in a *ΔallC* genetic background

Strains and primers used in this study are described in Table 1. Deletion mutants of *allW*, *allB* and *allC* were generated from the parental strain (*E. coli* K-12 strain BW25113) using lambda red recombination^22^. Briefly, to generate each mutant, a FLP recognition site (FRT)-flanked kanamycin resistance gene (*km*) was amplified from the template plasmid pKD4 using a pair of specific mutant oligonucleotides, gel purified, and then integrated into the genome to replace the target gene. The *km* gene was subsequently eliminated (leaving an 85-bp FRT sequence) using plasmid pCP20 that bears the FLP recombinase. The replacement of each target gene by the FRT-flanking *km* gene and subsequent removal of the *km* gene were confirmed by colony PCR and DNA sequencing, yielding deletion mutants Δ*allW,* Δ*allB* and Δ*allC,* respectively (Table 1).

To construct *allW–allC* and *allB–allC* double deletion mutants using similar methods as above, the *allC* gene was deleted in strain Δ*allW* and strain Δ*allB* individually following the same procedure, yielding double deletion mutants Δ*allW–*Δ*allC* and Δ*allB–*Δ*allC,* respectively.

### Docking methods

Glycerate-2 kinase (GlxK) (homology model, based on 58% sequence identity with PDB id 3CWC:A) was docked to monomeric *E. coli* allantoinase (PDB id 3E74:A) using CPORT^31^ predicted residues as restraints in HADDOCK docking^32^. This resulted in 3 high-scoring clusters of complexes, or complexes with a HADDOCK score of > 1.0 standard deviations away from the average across the top 200 complexes. Two of these three were nearly identical upon manual inspection, and the higher-scoring of the two was selected for the docking model. This complex was overlaid onto tetrameric allantoinase B (PDB id 3E74:A-D). The primary interactions, those that were docked 1:1 using HADDOCK, are between GlxK and AllB subunits A and D, while the secondary or neighbouring interactions are between GlxK and AllB subunits B and C. Visualization of key residues was performed in order to investigate potential mechanisms for the observed activation of AllB by GlxK. Lysine residues 2, 11, and 18 in GlxK, which are suspected to play a role in the carboxylation of K146 at the active site of AllB, were highlighted in Pymol (https://pymol.org/) (Fig. 6). The Consurf analysis was carried out on the consurf server at https://consurf.tau.ac.il/ .

### GlxK activity

Glycerate kinase produces ADP, detected by assay with coupling enzymes, pyruvate kinase (PK) and lactate dehydrogenase (LDH). Oxidation of NADH was followed by measuring the decrease in the absorbance at 340 nm. We added GlxK (10 ng) to 100 μl of a reaction mixture containing 50 mM Tris–HCl buffer, pH 7.4, 10 mM MgSO_4_, 0–450 μM glycerate, 1.2 mM ATP, 1.2 mM PEP, 0.3 mM NADH, and 1.2 U each of PK and LDH. Reaction rates were compared to controls in which glycerate was absent. The kinase activity detection kit was used as described^33^.

### AllB activity

The AllB_Mn and AllB_Zn purified protein was assayed by measuring the hydrolysis of allantoin as described^20,34^. The assay mixture contained 0.1 M Tris–HCl, pH 8.0, 0.1 × PBS buffer, 2% glycerol and 0.2 mM MnSO_4_ (0.1 mM ZnSO_4_). Allantoin was freshly dissolved immediately before use, and the activity was measured in 100 μl of a reaction mixture containing 0–15 mM allantoin. For the MnSO_4_ purified AllB_Mn (15 nM), 0.2 mM MnSO_4_ was added to the assay mixture with 2% glycerol and 0.1 × PBS. To determine the effect of GlxK on AllB activity, 130 nM GlxK was added to the assay mixture. The observed reaction rates, calculated using an allantoate extinction coefficient of 0.0261 mM^−1^ cm^−1^ at 257 nm, were compared with those for the control sample.

### AllB, AllW and GlxK co-immunoprecipitation

*glxK* and *allW* were expressed from a clone of the ASKA overexpression library (in plasmid pCA24N, Genbank:AB052891) using an IPTG-inducible promoter and chloramphenicol resistance to yield His_6_ tagged proteins^35^. These plasmids were isolated (using a Qiagen plasmid Miniprep kit), and then transformed into strains synthesising AllB. Labelled strains with an endogenously FLAG-tagged gene under the control of its native promoter (marked with kanamycin), were expressed in *E. coli* strain DY330, with the resulting transformants selected on kanamycin (50 μg/ml) and chloramphenicol (34 μg/ml). The positive transformants were grown at 32 °C in 50 ml of Luria broth (LB) media with antibiotic selection until the cells reached an OD_600_ of 0.5. Then 1 mM of IPTG was added for induction for 2 h at 32 °C, and glyoxylate or allantoin was added to a final concentration of 0.7 mM or 2 mM in LB, respectively, followed by incubation for 2 more hours. The harvested cells were centrifuged again at 4000 × g for 15 min, and they were immediately lysed by sonication, followed by centrifugation at 12,000 × g for 20 min. The resulting supernatant was used for immunoprecipitation with anti-FLAG-tag magnetic beads using µMACS kits (Miltenyi Biotec) to capture the Flag-epitoped AllB and AllW proteins. After samples of the Co-IP proteins were eluted from the anti-Flag-tag magnetic beads, aliquots were applied to 10% SDS-PAGE for subsequent immunoblotting as described previously^36^. The membranes were probed with HRP-conjugated monoclonal (α-FLAG, α-His epitope) antisera and were visualized by chemiluminescence (Pierce).

### Ethical approval

This work does not contain any studies with animals performed by any of the authors.

## Supplementary Information

Supplementary Information 1.

Supplementary Information 2.

Supplementary Information 3.

## Data Availability

All data generated or analyzed during this study are included in this published article (and its supplementary information files).
