# Supplementary material for: E. coli allantoinase is activated by the downstream metabolic enzyme, glycerate kinase, and stabilizes the putative allantoin transporter by direct binding
Source: Sci Rep. 2023 May 5;13:7345. doi: 10.1038/s41598-023-31812-4 (PMC10163214; doi:10.1038/s41598-023-31812-4)
Supplement: Supplementary file 1 — Supplementary Information 1. [file 41598_2023_31812_MOESM1_ESM.pptx]

## Slide 1
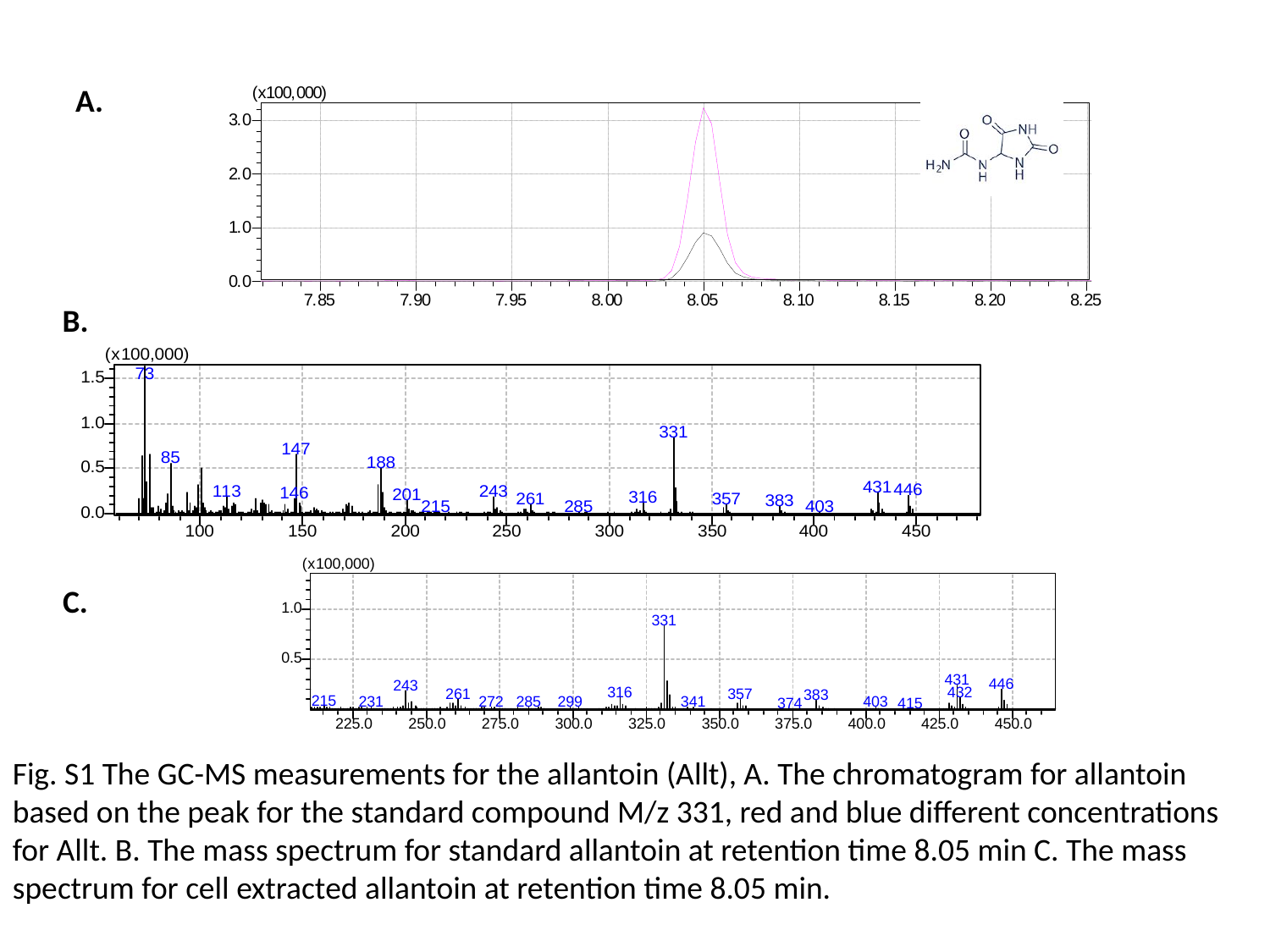

A.
B.
C.
Fig. S1 The GC-MS measurements for the allantoin (Allt), A. The chromatogram for allantoin based on the peak for the standard compound M/z 331, red and blue different concentrations for Allt. B. The mass spectrum for standard allantoin at retention time 8.05 min C. The mass spectrum for cell extracted allantoin at retention time 8.05 min.
