# Supplementary material for: E. coli allantoinase is activated by the downstream metabolic enzyme, glycerate kinase, and stabilizes the putative allantoin transporter by direct binding
Source: Sci Rep. 2023 May 5;13:7345. doi: 10.1038/s41598-023-31812-4 (PMC10163214; doi:10.1038/s41598-023-31812-4)
Supplement: Supplementary file 2 — Supplementary Information 2. [file 41598_2023_31812_MOESM2_ESM.pdf]

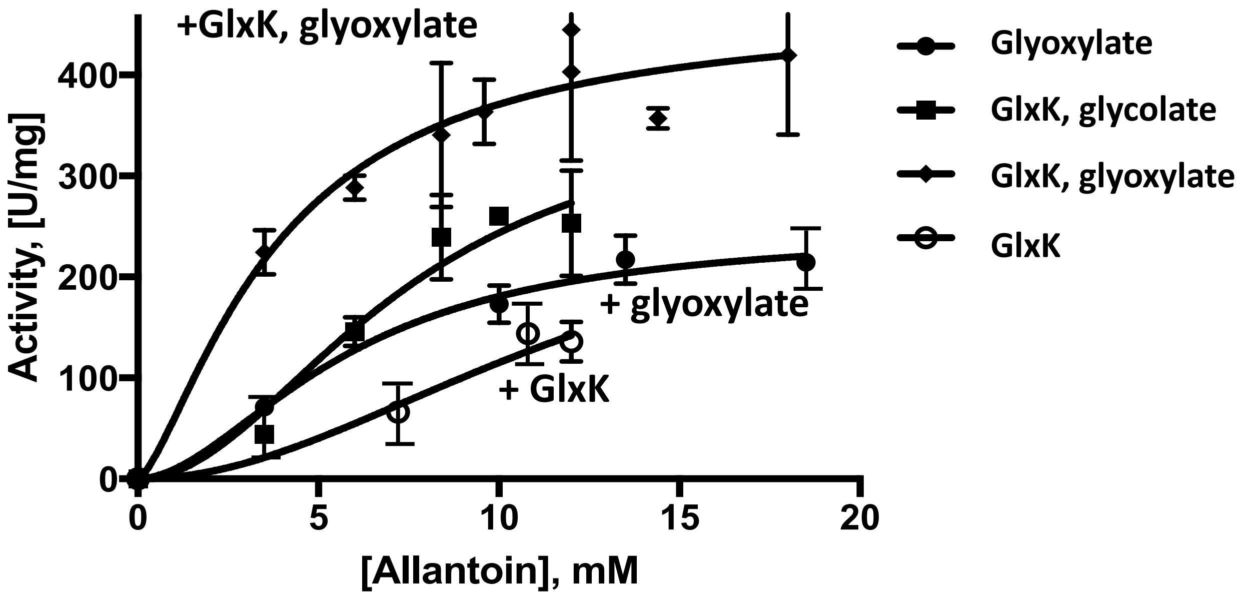

Fig. S5. The the activity of AllB was measured as a function of the allantoin concentration in the presence or the absence of GlxK (2.5 μM) and glyoxylate, units are presented in μmoles/min/mg, protein.
