# Supplementary material for: E. coli allantoinase is activated by the downstream metabolic enzyme, glycerate kinase, and stabilizes the putative allantoin transporter by direct binding
Source: Sci Rep. 2023 May 5;13:7345. doi: 10.1038/s41598-023-31812-4 (PMC10163214; doi:10.1038/s41598-023-31812-4)
Supplement: Supplementary file 3 — Supplementary Information 3. [file 41598_2023_31812_MOESM3_ESM.pdf]

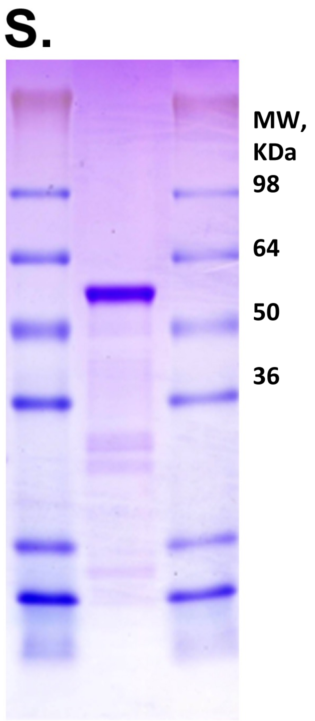

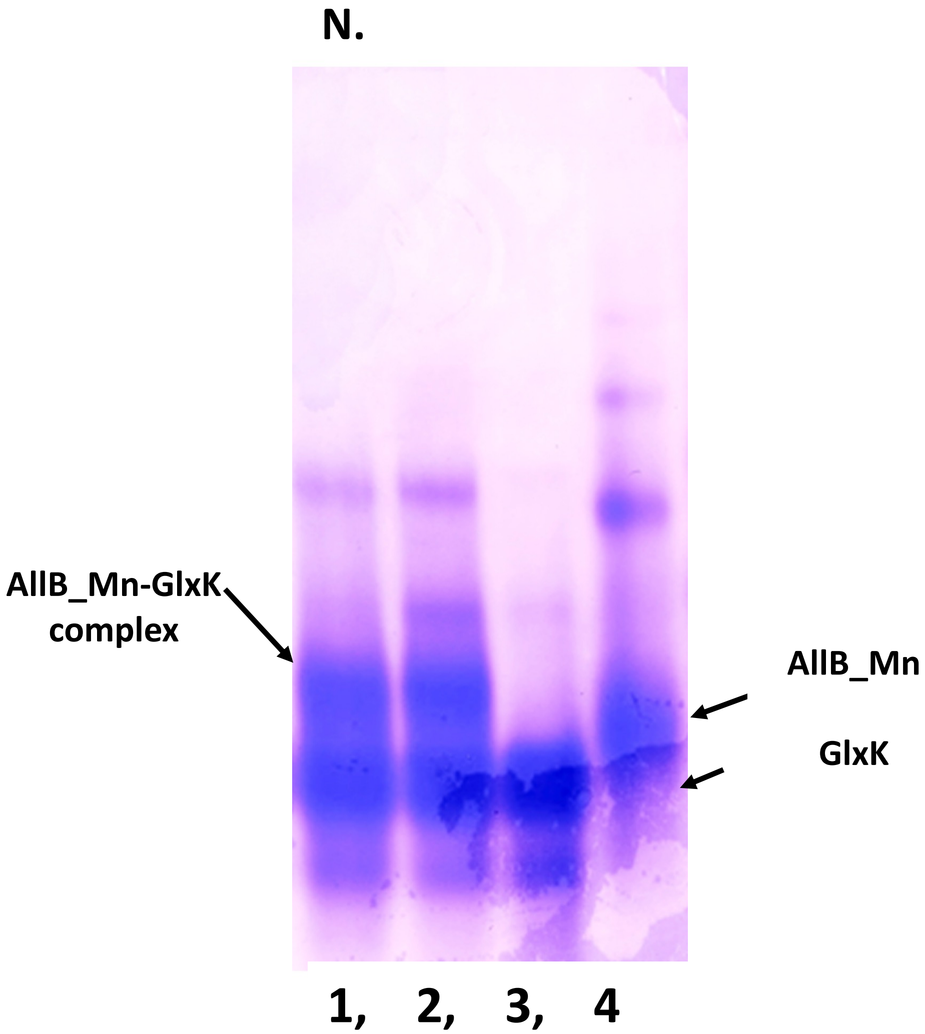

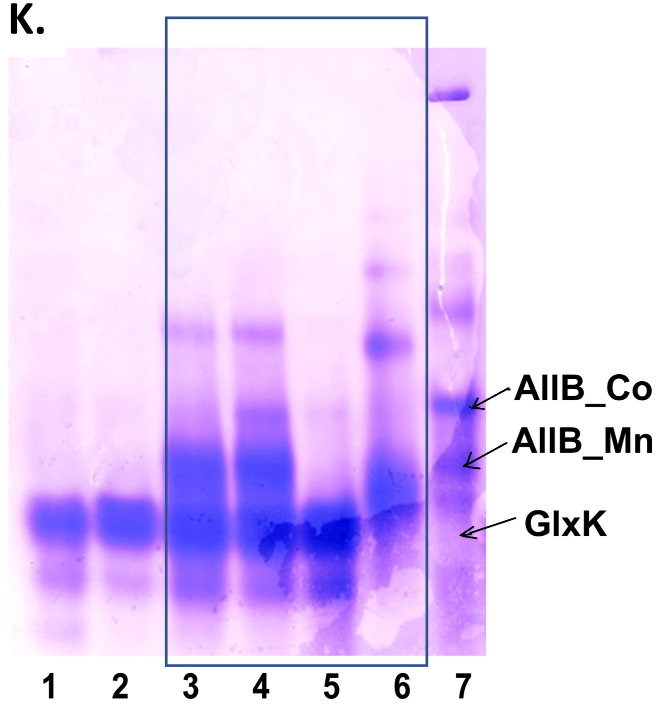

Fig.S2

S. The purified AllB His-tagged protein used for the kinetics experiments. **B**. Native gel electrophoresis for AllB and GlxK His-tagged purified proteins. Lanes 1, 2-AllB_Mn in complex with GlxK is marked by arrow, 3– GlxK, 4 - AllB_Mn purified proteins. K. The original native gel, AllB_Co, AllB produced with $CoCl_2$

Fig. S3 Full blot for Fig. 3A,B (Flag-antibody, marked with the frame)

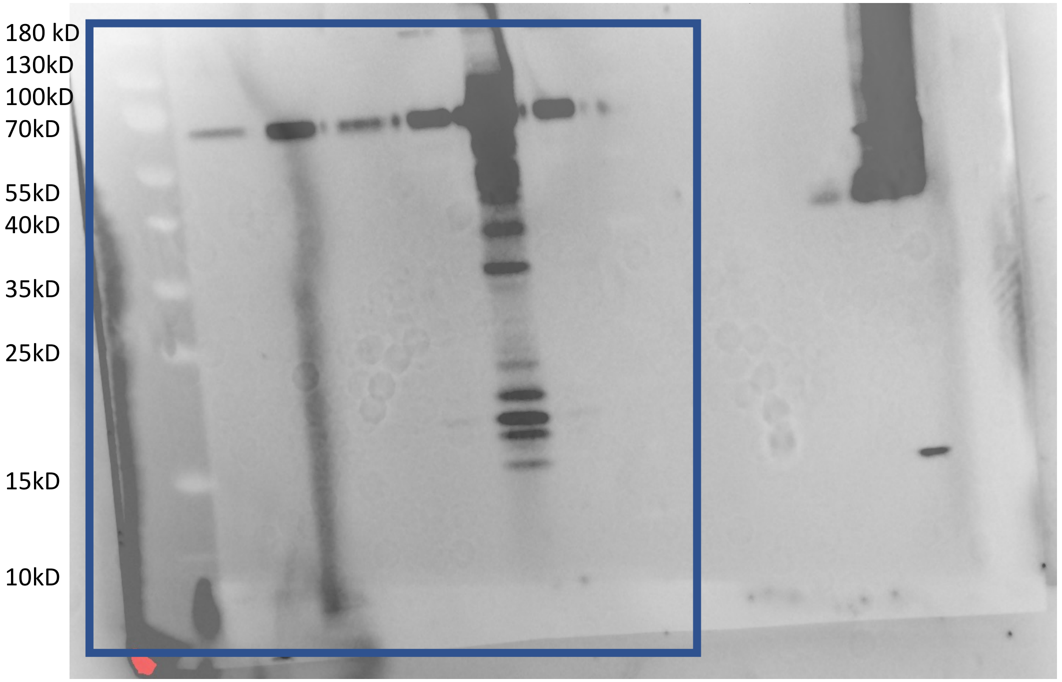

Fig. S4 Full blot for Fig. 3C, His antibody (same membrane, marked with the frame)

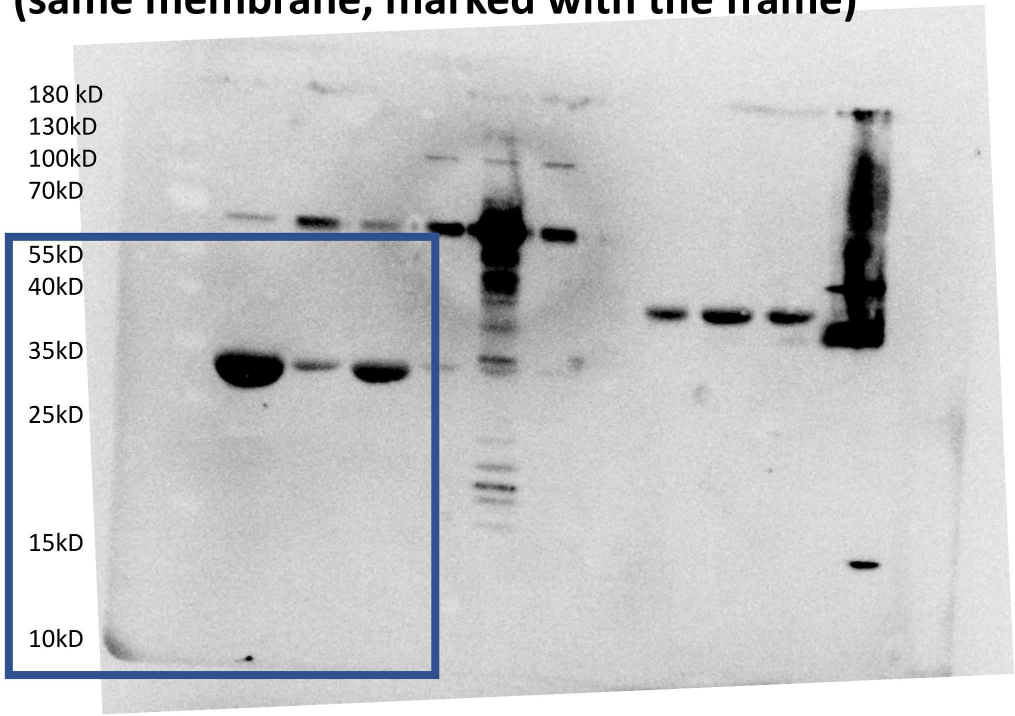

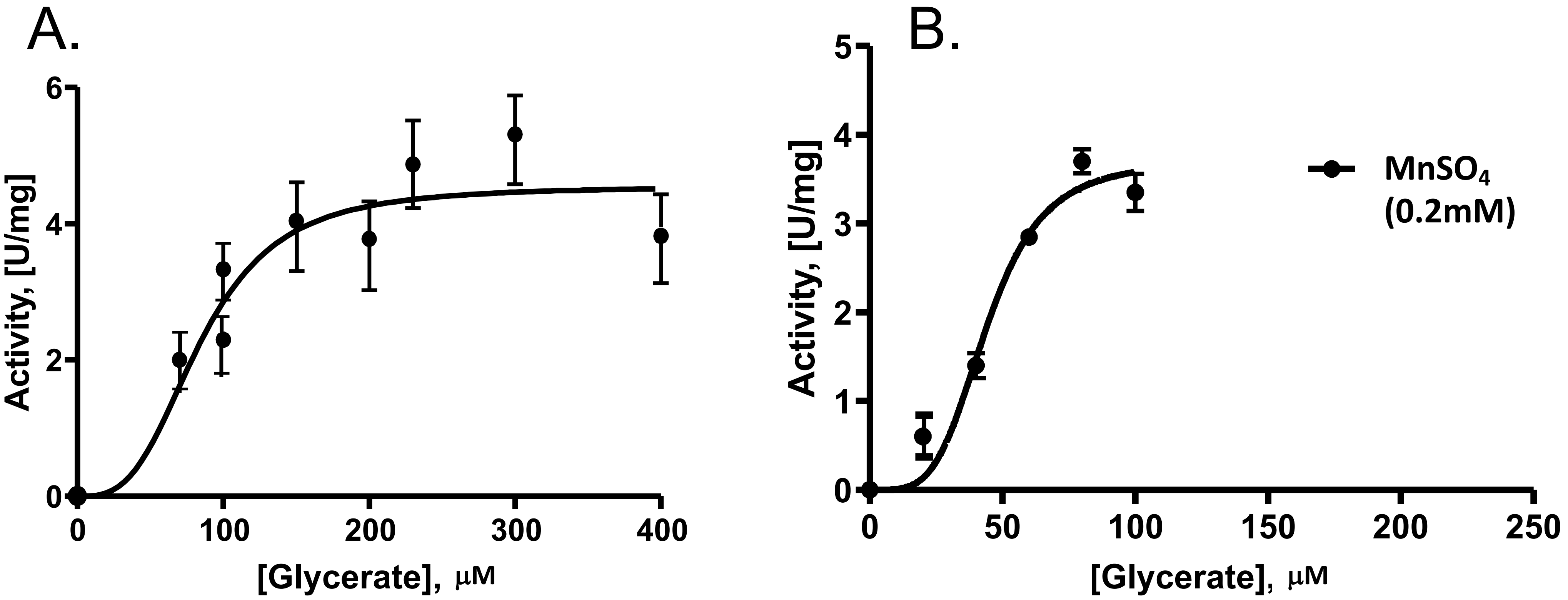

Fig. S6. The the activity of GlxK was measured as a function of the glycerate concentration in the presence or the absence of 200 μM $MnSO_4$, units are presented in μmoles/min/mg, protein.
